# Adiponectin-Mediated Analgesia and Anti-Inflammatory Effects in Rat

**DOI:** 10.1371/journal.pone.0136819

**Published:** 2015-09-09

**Authors:** Tommaso Iannitti, Annette Graham, Sharron Dolan

**Affiliations:** Department of Life Sciences, School of Health and Life Sciences, Cowcaddens Road, Glasgow Caledonian University, Glasgow, G4 0BA, United Kingdom; University of Texas at Dallas, UNITED STATES

## Abstract

The adipose tissue-derived protein, adiponectin, has significant anti-inflammatory properties in a variety of disease conditions. Recent evidence that adiponectin and its receptors (AdipoR1 and AdipoR2) are expressed in central nervous system, suggests that it may also have a central modulatory role in pain and inflammation. This study set out to investigate the effects of exogenously applied recombinant adiponectin (via intrathecal and intraplantar routes; 10–5000 ng) on the development of peripheral inflammation (paw oedema) and pain hypersensitivity in the rat carrageenan model of inflammation. Expression of adiponectin, AdipoR1 and AdipoR2 mRNA and protein was characterised in dorsal spinal cord using real-time polymerase chain reaction (PCR) and Western blotting. AdipoR1 and AdipoR2 mRNA and protein were found to be constitutively expressed in dorsal spinal cord, but no change in mRNA expression levels was detected in response to carrageenan-induced inflammation. Adiponectin mRNA, but not protein, was detected in dorsal spinal cord, although levels were very low. Intrathecal administration of adiponectin, both pre- and 3 hours post-carrageenan, significantly attenuated thermal hyperalgesia and mechanical hypersensitivity. Intrathecal administration of adiponectin post-carrageenan also reduced peripheral inflammation. Intraplantar administration of adiponectin pre-carrageenan dose-dependently reduced thermal hyperalgesia but had no effect on mechanical hypersensitivity and peripheral inflammation. These results show that adiponectin functions both peripherally and centrally at the spinal cord level, likely through activation of AdipoRs to modulate pain and peripheral inflammation. These data suggest that adiponectin receptors may be a novel therapeutic target for pain modulation.

## Introduction

Adiponectin, an anti-inflammatory adipokine lowered in plasma in individuals with obesity, is known to have significant anti-inflammatory, anti-atherogenic, anti-apoptotic, and insulin-sensitising properties [1, 2]. While a number of experimental studies have established a key role for adiponectin as an anti-inflammatory agent in relation to obesity and cardiovascular disease, in part through inhibition of inflammatory cytokines [3–5], its role in inflammatory pain is largely unknown. Evidence that intra-articular administration of adiponectin reduces the severity of collagen-induced arthritis in mouse [6], through modulation of tumour necrosis factor (TNF)-α, interleukin (IL)-1β, IL-6, and matrix metalloproteinase (MMP)-3 in joint tissue, suggested that adiponectin may play a role in pain pathophysiology. This is supported by studies showing a dysregulation of serum adiponectin levels in patients with chronic daily headache [7], variant angina [8], and coronary spastic angina, a form of chest pain [9].

Although first reported to be exclusively derived from adipocytes, adiponectin has since been found in a range of tissues such as skeletal muscle, liver and osteoblasts [10], chicken and murine brain [11, 12], and human pituitary gland [13, 14]. The seven-transmembrane domain adiponectin receptors AdipoR1 and AdipoR2 are also widely distributed in brain [13–17], where they are involved in regulation of energy homeostasis, food intake and autonomic functions [10]. Recent work in this laboratory reported that adiponectin, AdipoR1 and AdipoR2 mRNAs are expressed in rat spinal cord [18], suggesting that a local adiponectin signalling system may exist at this level. Findings that adiponectin mRNA was down-regulated in spinal cord from obese Zucker rats [18], strengthened the hypothesis that adiponectin may have a modulatory role in inflammatory pain processing. The aim of this study was to investigate whether adiponectin has anti-inflammatory and anti-hyperalgesic effects when administered centrally at the spinal level, compared to peripheral administration, in a well characterised rodent model of peripheral inflammation.

## Materials and Methods

### Animals

All studies were approved by the Glasgow Caledonian University’s Animal Welfare and Ethical Review Body and all procedures were performed in accordance with the UK Animal Scientific Procedures Act (1986). Animals were treated in accordance with the Ethical Guidelines for Investigations of Experimental Pain in Conscious Animals issued by the International Association for the Study of Pain. Adult male Wistar rats aged 10–12 weeks (230–360 g; n = 101) were bred in house, maintained in group cages (with 2 littermates) on saw dust bedding, and subjected to a 12-hour light/12-hour dark cycle with food and water provided *ad libitum*. Animals were killed immediately after the experiment by intraperitoneal administration of sodium pentobarbital (5 mg/100 g; JM Loveridge PLC, Southampton, UK). All data presented are in accordance with the ARRIVE guidelines for reporting experiments involving animals [19].

### Expression of adiponectin, AdipoR1 and AdipoR2 in spinal cord

Lumbar spinal cord was collected from a group of adult male Wistar rats (n = 10/group), after intraplantar (i.pl.) injection of carrageenan (3%; 50 μl) or saline (50 μl, i.pl.) into the left hindpaw, and euthanized with pentobarbital sodium (100 mg/kg; i.p.; Pharmasol, JM Loveridge PLC, Southampton, UK) 6 hours post-injection (the time of maximum hyperalgesia and paw oedema) [20]. Dorsal spinal cord tissues from these animals were hemisected into left (ipsilateral) and right (contralateral) sections and stored at -80°C, to be processed for use in real-time PCR or Western blotting. Total RNA was extracted using an RNeasy extraction kit (Qiagen, UK), and cDNA prepared as described previously [18]. Levels of adiponectin, AdipoR1 and AdipoR2 mRNA were measured relative to cyclophilin as described [18]; specific sequences for primers and fluorescent probes (FAM/TAMRA) are shown in Table 1. Results are expressed as ratio to cyclophilin, using the 2^-ΔCt^ comparison method (Applied Biosystems; User Bulletin 2).

**Table 1:** Real-Time PCR probe, forward and reverse primer sequences for detection of rat cyclophilin, adiponectin, adiponectin receptor 1 (AdipoR1) and adiponectin receptor 2 (AdipoR2).

	Forward Primer	Reverse Primer	Probe
**Cyclophilin**	AGGGTTCCTCCTTTCACAGAATTAT	GCCACCAGTGCCATTATGG	CCACCCTGGCACATGAATCCTGG
**Adiponectin**	CCCCTGGCAGGAAAGGA	TCCAGCCCTACGCTGAATG	CCCGGAGAAGCCGCTTACATGTATCA
**AdipoR1**	CTACATGGCCACAGACCACCTAT	TGTGGATGCGGAAGATGCT	CCCTCCTTCCGGGCTTGCTTCA
**AdipoR2**	CTATATCACAGGAGCTGCCCTCTAC	ATGTCACATTTGCCAGGAAAGA	CGGCCCGTATCCCTGAGCGC

Proteins were extracted from dorsal spinal cord tissues (ipsilateral and contralateral tissues were pooled) from healthy adult male Wistar rats to confirm expression of adiponectin, AdipoR1 and AdipoR2 proteins in this tissue, using radioimmunoprecipitation (RIPA) buffer according to protocol described by Dolan et al. [21]. Proteins were also extracted from positive control tissues: brain, liver and adipocyte cell lysates (Santa Cruz Biotechnology, Inc. USA). All protein lysates were diluted in loading buffer (NuPage LDS sample buffer, 1 × final concentration (Invitrogen, UK); 0.05 M DTT) to a final concentration of 2 mg ml^-1^ and incubated for 10 min at 90°C. Diluted protein lysates (30 μg) and protein molecular weight markers (SeaBlue Plus 2 Pre-Stained Standard; Invitrogen, UK) were loaded onto NuPage Novex 4–12% Bis-Tris gels (Invitrogen, UK), and run for 50 min at 200 V. Proteins were transferred onto PVDF membranes using the iBlot semi-dry blot device (Invitrogen, UK). Membranes were blocked in 0.1 M PBS; 10% skimmed milk proteins; 0.1% Tween 20 for 1 h at room temperature. Following blocking, membranes were incubated overnight at 4°C with either adiponectin (rabbit polyclonal; 1:1000; Abcam, UK), AdipoR1 (rabbit polyclonal; 1:1000; Santa Cruz Biotechnology, Inc. USA) or AdipoR2 (goat polyclonal; 1:1000; Santa Cruz Biotechnology, Inc. USA) primary antisera diluted in blocking buffer. Immunoblots were then washed for 3 × 15 min in 0.1 M PBS; 0.1% Tween 20 prior to incubation for 1 h at room temperature in infrared fluorescence, IRDye secondary antibodies (800CW Goat anti-Rabbit IgG or 680RD Donkey anti-Goat IgG; 1:10,000; LI-COR Biosciences Ltd., UK) solution diluted in blocking buffer. Immunoblots were washed for 3 × 15 min in 0.1 M PBS; 0.1% Tween 20 and visualised on the Odyssey infrared imaging system (LI-COR Biosciences Ltd., UK).

### Behavioural testing and measurement of paw oedema

Thermal and mechanical nociceptive thresholds and paw oedema were measured as described previously [18]. Baseline response thresholds to thermal and mechanical stimulation of both hindpaws were measured at time 0, immediately before i.pl. injection of carrageenan (3%; 50 l) into the left hindpaw, using the Ugo Basile Thermal Plantar Test and a dynamic plantar aesthesiometer (Ugo Basile Model 7370; Linton Instrumentation, UK), then at 2, 4, 6, 8 and 24 hours after. Each rat received between 3 and 6 trials per hindpaw, with at least 30 seconds separating trials. The mean of all trials for each hindpaw was calculated and expressed as latency to withdrawal in seconds or force in grams. Paw volume was measured using a plethysmometer (Ugo Basile, Italy), and data represented as the maximum change in paw volume from baseline (Time 0).

### Drug treatment

Rat recombinant adiponectin (aa 16–244; Soluble (rat) (rec.) (FLAGR); ALX-201-303-C050) produced in Human Embryonic Kidney Cell Line 293 and presented in liquid form as a 0.2 μm-filtered solution in 30 mM TRIS-HCl (pH 8.5) was purchased from Alexis Biochemicals (Enzo Life Sciences, Inc., USA). Aliquots were stored at -20°C until required. To investigate central effects, adiponectin (10, 100 and 1000 ng) or drug-vehicle (saline) was administered intrathecally (i.t.) to adult male Wistar rats (n = 5-10/group; 250–330 g) in a volume of 15 μl either 5 minutes pre-carrageenan or 3 hours post-carrageenan, under light anaesthesia maintained with 2% isoflurane and O_2_ (2L/min) delivered via a nose cone. Intrathecal injections were made through L5- L6 vertebrae using a 30 gauge needle. In the second study, adult male Wistar rats (n = 5-6/group; 250–330 g) received an i.pl. injection of adiponectin (100, 1000 and 5000 ng) or drug-vehicle (saline) in a volume of 50 μl into the left (ipsilateral) hindpaw either 5 minutes pre-carrageenan or 3 hours post-carrageenan.

### Statistical analysis

Data are represented as the mean ± standard error of mean (SEM). Threshold response data were analysed using two-way mixed factorial ANOVA (SPSS, v17, U.S.A), where treatment is the between-subject factor and time post-treatment is the within-subjects (repeated measures) factor. Mean differences were calculated using simple effects analyses applying Bonferroni adjustment factor for multiple comparisons. The maximum effect (E_max_%) was also calculated for each animal as the maximum change in response or paw volume (after treatment) from baseline. These data were analysed using the one-way ANOVA with post-hoc Tukey’s tests (SPSS, v17, U.S.A).

## Results

### Expression of adiponectin, AdipoR1 and AdipoR2 in spinal cord

**Fig 1:**
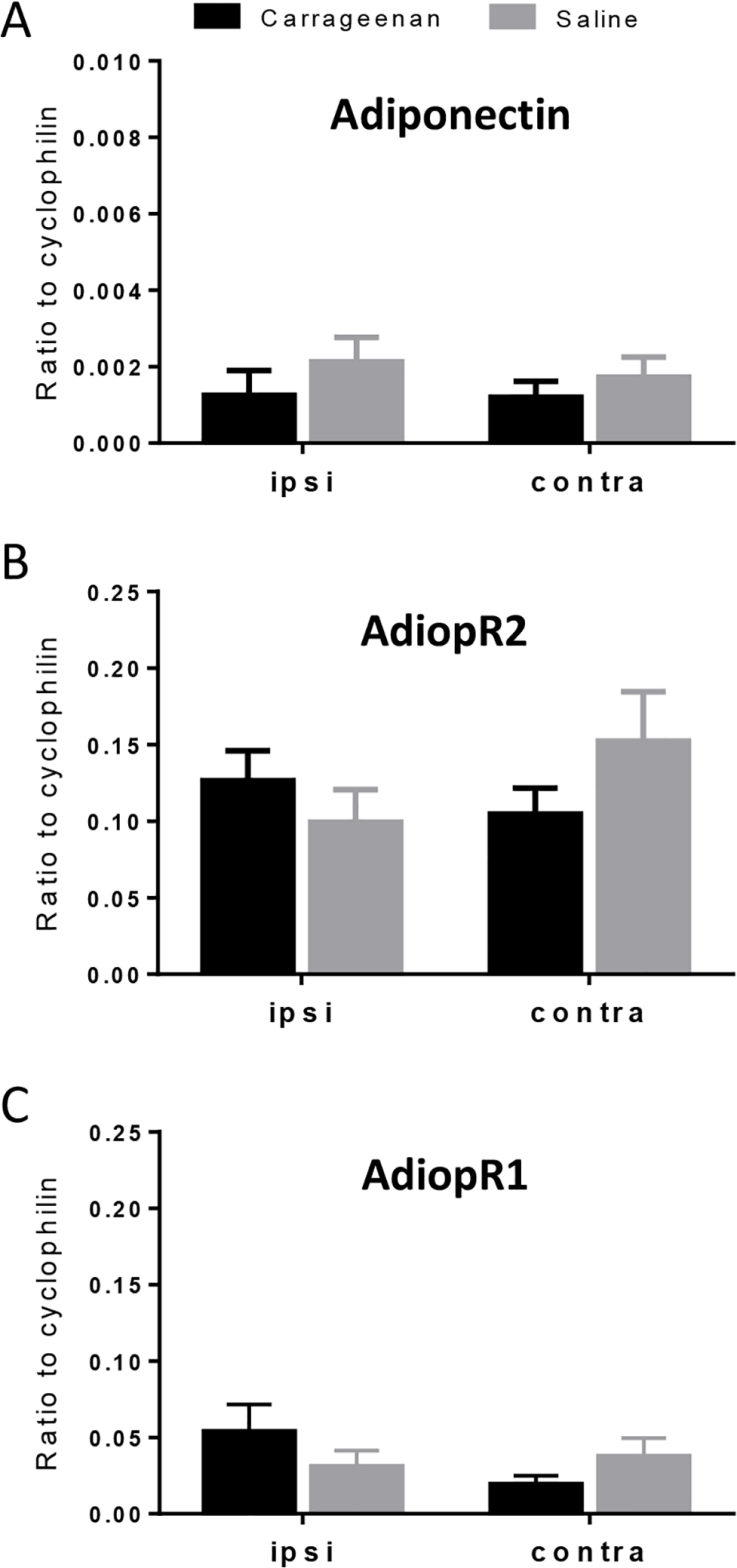
Real-time PCR analysis of adiponectin (A), AdipoR1 (B) and AdipoR2 (C) mRNA expression in ipsilateral (ipsi) and contralateral (contra) spinal cord collected 6 hours after injection of carrageenan (3%; 50 μl, i.pl.) or saline (50 μl, i.pl.) into the left hindpaw (n = 10 per group). Expression of target mRNA levels are expressed relative to the housekeeping gene cyclophilin using the 2^-ΔCt^ calculation. Data are presented as the mean ± SEM.

**Fig 2:**
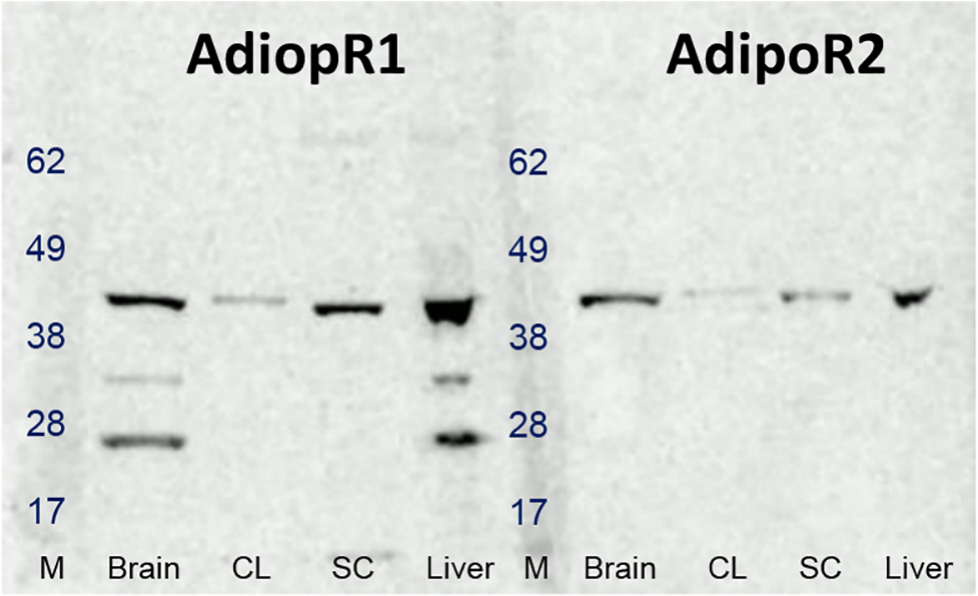
Expression of AdipoR1 and AdipoR2 proteins in rat spinal cord (SC), brain, liver and cell lysate (CL) detected by Western blotting. Photomicrograph shows expression of AdipoR1 and AdipoR2 at 45 kDa, as expected, in a representative control animal. Protein molecular weights are indicated in the size marker (M) lanes.

Adiponectin, AdipoR1 and AdipoR2 mRNA were constitutively expressed in spinal cord. AdipoR1 mRNA was more abundantly expressed than AdipoR2 (approx. 4-fold higher; P < 0.001). Adiponectin mRNA levels were very low in spinal cord, and in some cases undetectable. Following carrageenan-induced inflammation, no change was detected in levels of adiponectin, AdipoR1 or AdipoR2 mRNA in spinal cord (Fig 1). Western blot analyses of protein extracts prepared from spinal cord homogenates revealed AdipoR1 and AdipoR2 antibody labelled bands at approximately 45 kDa, as expected (Fig 2). Both receptors were also detected in all three positive control tissues, with the highest expression seen in brain and liver for both. Adiponectin protein was undetectable in spinal cord by Western blotting.

### The effect of intrathecal adiponectin on carrageenan-induced thermal hyperalgesia, mechanical hypersensitivity and paw oedema

**Fig 3:**
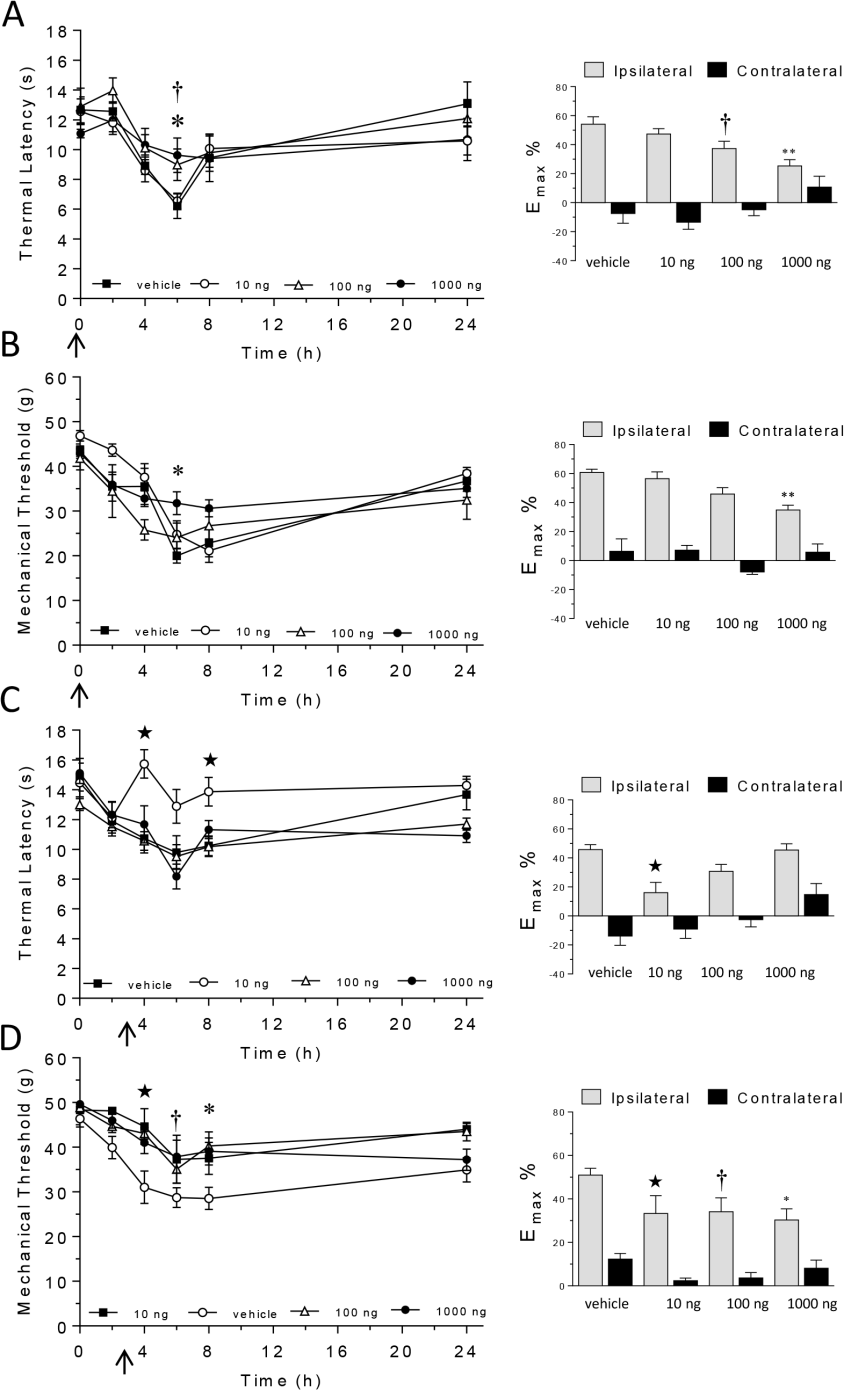
The effect of intrathecal administration of adiponectin (10, 100, 1000 ng) or drug-vehicle pre- (A, B) or 3 hours (C, D) post-carrageenan on thermal hyperalgesia (A, C) and mechanical hypersensitivity (B, D). Graphs in left-hand column show thermal response latencies (s) and mechanical response thresholds (force/g) at baseline (time 0) and 2, 4, 6, 8 and 24 hours post-carrageenan. Graphs in the right-column show the maximum change in thermal latency (A, C) and mechanical threshold (B, D) from baseline in ipsilateral and contralateral hindpaws represented as a percentage (E_max_%). Significant attenuation of thermal hyperalgesia/mechanical hypersensitivity: ★ *P* < 0.05 for 10 ng vs. drug-vehicle; † *P* < 0.05 for 1000 ng vs. drug-vehicle; * *P* < 0.05, ** *P* < 0.01 for 5000 ng vs. drug-vehicle. Arrow represents time of adiponectin/drug-vehicle injection.

Carrageenan induced significant thermal hyperalgesia in the injected paw in drug-vehicle treated animals (Fig 3A and 3C); thermal latency was significantly reduced from baseline at 4 (p < 0.05 vs. baseline responses), 6 and 8 hours (both p < 0.01 vs. baseline responses). Intrathecal pre-administration of adiponectin (100, 1000 ng) significantly attenuated thermal hyperalgesia at 6 hours (p < 0.05 vs. drug-vehicle; Fig 3A), while post-administration of low dose adiponectin (10 ng) significantly attenuated thermal hyperalgesia at 4 and 8 hours (p < 0.01 vs. drug-vehicle; Fig 3C).

Carrageenan induced significant mechanical hypersensitivity in the injected paw in drug-vehicle treated animals (Fig 3B and 3D); mechanical thresholds were significantly reduced from baseline at 6, 8 (both p < 0.01 vs. baseline responses) and 24 hours post-carrageenan injection (p < 0.05 vs. baseline responses). Intrathecal pre-administration of high dose of adiponectin (1000 ng), and post-administration of all three other doses investigated (10, 100 and 1000 ng) significantly attenuated carrageenan-induced mechanical hypersensitivity (all p < 0.05 vs. drug-vehicle; Fig 3B and 3D).

Carrageenan induced significant paw oedema in the injected paw in drug-vehicle treated animals, maximum 6 hours post-carrageenan [maximum change in paw volume data (E_max_%) are shown in Table 2]. Pre-administration of intrathecal adiponectin had no effect on paw oedema; in contrast, intrathecal administration of 1000 ng adiponectin 3 hours post-carrageenan significantly reduced paw oedema (P < 0.05 vs. drug-vehicle).

Nociceptive responses and paw volume remained unchanged in the contralateral paw for the duration of the study.

**Table 2:** The effect of intrathecal (i.t.) or intraplantar (i.pl.) administration of adiponectin (10, 100, 1000 or 5000 ng) or drug-vehicle injected either pre- or 3 hours post-carrageenan on paw oedema. Data are represented as the maximum change in paw volume (E_max_(%)) after carrageenan injection from baseline.

E_max_ (%)					
	Dose	Pre-carrageenan	*n*	Post-carrageenan	*n*
**i.t.**	Drug-vehicle	61.2 ± 3.5	*6*	60.4 ± 3.7	*9*
	10 ng	58.0 ± 3.0	*6*	55.0 ± 4.0	*6*
	100 ng	61.3 ± 7.0	*5*	55.2 ± 5.7	*9*
	1000 ng	65.8 ± 3.6	*5*	26.9 ± 3.2*	*7*
**i.pl.**	Drug-vehicle	61.3 ± 3.5	*6*	74.4 ± 6.0	*6*
	100 ng	51.4 ± 5.6	*6*	76.8 ± 3.2	*6*
	1000 ng	62.7 ± 13.6	*5*	88.6 ± 7.2	*6*
	5000 ng	68.7 ± 12.7	*6*	95.3 ± 3.2	*6*

* *P* < 0.05 vs. drug-vehicle.

### The effect of intraplantar adiponectin on carrageenan-induced thermal hyperalgesia, mechanical hypersensitivity and paw oedema

**Fig 4:**
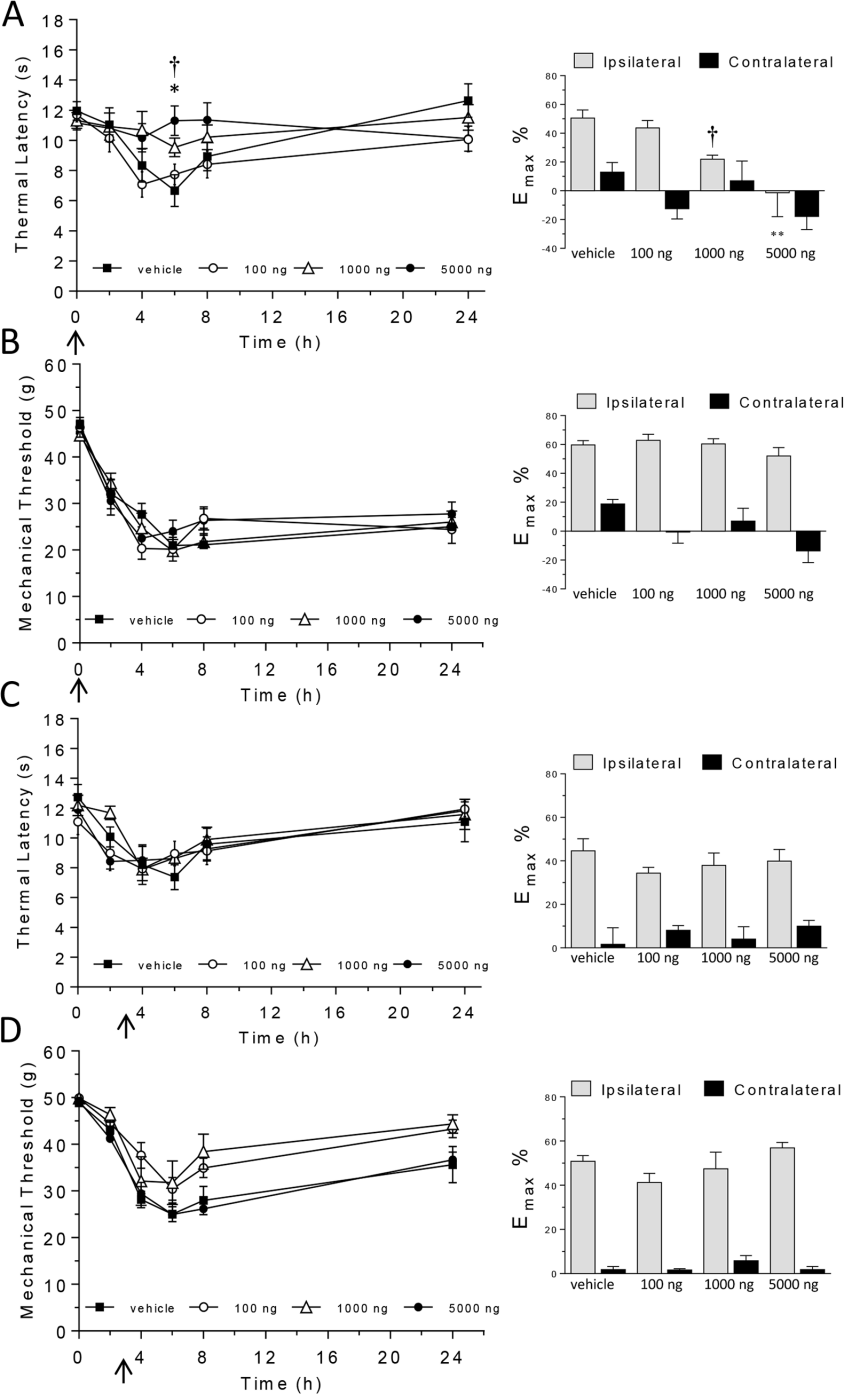
The effect of intraplantar administration of adiponectin (100, 1000, 5000 ng) or drug-vehicle pre- (A, B) or 3 hours (C, D) post-carrageenan on thermal hyperalgesia (A, C) and mechanical hypersensitivity (B, D). Graphs in left-hand column show thermal response latencies (s) and mechanical response thresholds (force/g) at baseline (time 0) and 2, 4, 6, 8 and 24 hours post-carrageenan. Graphs in the right-column show the maximum change in thermal latency (A, C) and mechanical threshold (B, D) from baseline in ipsilateral and contralateral hindpaws represented as a percentage (E_max_%). Significant attenuation of hyperalgesia: † *P* < 0.05 for 1000 ng vs. drug-vehicle; * *P* < 0.05, ** *P* < 0.01 for 5000 ng vs. drug-vehicle. Arrow represents time of adiponectin/drug-vehicle injection.

Carrageenan induced significant thermal hyperalgesia (Fig 4B and 4D), mechanical hypersensitivity (Fig 4B and 4D) and paw oedema in the injected paw in animals treated with intraplantar drug-vehicle. Thermal latency was significantly reduced from baseline at 4 (p < 0.01 vs. baseline responses) and 6 hours (p < 0.05 vs. baseline responses), while mechanical thresholds were significantly reduced from baseline at 4, 6 (all p < 0.01 vs. baseline responses), 8 and 24 hours post-carrageenan (p < 0.05 vs. baseline responses). Paw oedema was maximum 8 hours post-carrageenan in drug-vehicle treated animals (p < 0.01 vs. baseline; maximum change in paw volume (E_max_%) data are shown in Table 2). Nociceptive responses and paw volume remained unchanged in the contralateral paw for the duration of the study.

Intraplantar pre-administration of adiponectin (1000, 5000 ng) significantly attenuated thermal hyperalgesia at 6 hours (p < 0.05 vs. drug-vehicle; Fig 4A), but had no effect on carrageenan-induced mechanical hypersensitivity or paw oedema at any time point. Intraplantar administration of adiponectin 3 hours post-carrageenan had no effect on thermal hyperalgesia (Fig 4C), mechanical hypersensitivity (Fig 4D) or paw oedema (Table 2).

## Discussion

The present study provides the first evidence of AdipoR1 and AdipoR2 protein expression in the rat spinal cord. Furthermore, results reveal novel evidence that application of adiponectin directly to the spinal cord inhibits inflammatory pain and attenuates carrageenan-induced peripheral inflammation. On the other hand, when adiponectin was administered directly into the paw, it was less effective, as only pre-administration of adiponectin was able to attenuate carrageenan-induced thermal hyperalgesia, but not mechanical hypersensitivity or paw oedema. This report is the first to show that adiponectin acts centrally at the spinal level to modulate both pain and peripheral inflammation. Further investigations confirmed our previous findings that adiponectin, and AdipoR1 and AdipoR2 mRNA are constitutively expressed in rat spinal cord [18], although no change was detected in levels of expression in response to carrageenan-induced inflammation at the time point measured. Previous studies have shown that the AdipoRs are expressed in various regions of the brain [10]; their presence in spinal cord, at mRNA and protein level, supports a functional role for adiponectin in this system.

Adiponectin is secreted into the circulation in three isoforms: low-molecular weight trimers, medium molecular weight hexamers, and high-molecular weight (HMW) multimers comprised of 4–6 trimers [22]. It also exists as a proteolytic cleavage fragment consisting of the globular C-terminal domain, known as globular adiponectin. HMW adiponectin is regarded as the major metabolically active form, while the central actions have been attributed to the hexameric and trimeric oligomers [23]. The rat recombinant adiponectin protein used in the current study mimics serum adiponectin by forming HMW and hexameric species, therefore presumed to be biologically active centrally. Adiponectin mediates its effect through binding AdipoR1 and AdipoR2, and a third receptor T-cadherin [24, 25]. While previous studies have shown that T-cadherin is present in rat spinal cord [26, 27]), this receptor was not investigated in the present study. AdipoR1 binds globular adiponectin with high affinity, while AdipoR2 shows intermediate affinity for both globular and full-length adiponectin. Both isoforms were expressed in spinal cord, AdipoR1 being the most abundantly expressed. AdipoR1 and AdipoR2 are also differentially expressed in various brain regions [11, 13, 17]. It is AdipoR1 that is thought to mediate the anti-inflammatory effects of adiponectin [28], and be primarily responsible for mediating hyperthermic effects of adiponectin in hypothalamus [29], suggesting that in spinal cord this receptor subtype may contribute to the anti-hyperalgesic and anti-inflammatory effects of adiponectin observed in this study.

It is well established that AdipoR1 and AdipoR2 are expressed in many peripheral tissues such as skeletal muscle, liver and adipose tissue [30–32], and evidence shows that these receptors are also expressed in peripheral nervous system tissues [33], strengthening the finding that peripheral administration of adiponectin may act at these receptors to inhibit the development of thermal hyperalgesia. Of interest, adiponectin was only effective via this route when administered pre-carrageenan (and not when given 3 hours after) and therefore before initiation of the sensitization. In contrast, spinal administration of adiponectin 3 hours post-carrageenan was able to reverse both thermal and mechanical hypersensitivity and paw oedema, suggesting that once peripheral and central sensitization are established, modulation of this pathway at the spinal cord level is key to regulating pain and inflammation.

The mechanism underlying a central mode of action for adiponectin has been investigated previously in the hypothalamic system, where intracerebroventricular injection of adiponectin was reported to regulate energy homeostasis and food intake [15, 29, 34]. Electrophysiological studies by Hoyda and coworkers [11] showed that adiponectin has both depolarizing and hyperapolarizing effects on distinct populations of neurons in the paraventricular nucleus of the hypothalamus. Further work by this group revealed that adiponectin exerts its hyperpolarizing effects by inhibiting non inactivating delayed rectifier potassium current (IK) [35], resulting in changes to membrane excitability and peptidergic release. We speculate that a similar modulatory role exists for adiponectin in spinal cord, especially with the growing evidence in the literature [36–39], and indeed in this lab (unpublished observations), that K^+^ channel activators produce anti-nociception in animal models of pain. Alternative mechanisms may exist, including adiponectin-mediated local regulation of transcription factor peroxisome proliferator-activated receptor-γ (PPARγ) [40], which has known anti-hyperalgesic properties [41–43], or inhibition of pro-inflammatory cytokines, such as TNF-α and IL-1β, which are induced in spinal cord following intraplantar injection of carrageenan [44, 45] and correlated with peripheral oedema [46]. Further work, however, is required to identify the mechanism underlying adiponectin-mediated anti-hyperalgesic effects in spinal cord.

In summary, the present study supports an anti-inflammatory role for adiponectin in central pathways, and for the first time, shows that this anti-inflammatory adipokine exerts anti-hyperalgesic effects, when administered centrally, likely through modulation of AdipoR1 or AdipoR2. This study gives rise to new perspectives for a future therapeutic role of adiponectin in inflammatory-related conditions and signals the need to better understand how adiponectin exerts its functions in the CNS.
